# Outpatient Cardiac Rehabilitation Closure and Home-Based Exercise Training During the First COVID-19 Lockdown in Austria: A Mixed-Methods Study

**DOI:** 10.3389/fpsyg.2022.817912

**Published:** 2022-02-15

**Authors:** Stefan Tino Kulnik, Mahdi Sareban, Isabel Höppchen, Silke Droese, Andreas Egger, Johanna Gutenberg, Barbara Mayr, Bernhard Reich, Daniela Wurhofer, Josef Niebauer

**Affiliations:** ^1^Ludwig Boltzmann Institute for Digital Health and Prevention, Salzburg, Austria; ^2^University Institute of Sports Medicine, Prevention and Rehabilitation, Salzburg, Austria; ^3^Research Institute of Molecular Sports Medicine and Rehabilitation, Paracelsus Medical University, Salzburg, Austria; ^4^Institute of Nursing Science and Practice, Paracelsus Medical University, Salzburg, Austria; ^5^Department of Health Promotion, CAPHRI, Maastricht University, Maastricht, Netherlands; ^6^REHA Zentrum Salzburg, Salzburg, Austria

**Keywords:** cardiovascular disease, exercise, interview, pandemics, quarantine

## Abstract

**Objective:**

To assess the impact of the closure of group-based cardiac rehabilitation (CR) training during the first COVID-19 lockdown in spring 2020 on patients’ physical activity, cardiorespiratory fitness, and cardiovascular risk, and to describe the patient experience of lockdown and home-based exercise training during lockdown.

**Design:**

Mixed methods study. Prospectively collected post-lockdown measurements were compared to pre-lockdown medical record data. Quantitative measurements were supplemented with qualitative interviews about the patient experience during lockdown.

**Setting:**

Outpatient CR centre in Salzburg, Austria.

**Participants:**

Twenty-seven patients [six female, mean (SD) age 69 (7.4) years] who attended weekly CR training sessions until the first COVID-19 lockdown in March 2020.

**Outcome Measure(s):**

Quantitative: exercise capacity (maximal ergometer test, submaximal ergometer training), cardiovascular risk (Framingham risk score, blood pressure, body mass index, lipids). Qualitative: individual semi-structured interviews.

**Results:**

Exercise capacity had significantly reduced from pre- to post-lockdown: mean (SD) power (W) in maximal ergometry 165 (70) vs. 151 (70), *p* < 0.001; submaximal ergometer training 99 (40) vs. 97 (40), *p* = 0.038. There was no significant difference in Framingham risk score and other cardiovascular risk factors. Qualitative data showed that almost all patients had kept physically active during lockdown, but 17 (63%) said they had been unable to maintain their exercise levels, and 15 (56%) felt their cardiorespiratory fitness had deteriorated. Many patients missed the weekly CR training and the motivation and sense of community from training together with others. Several patients stated that without professional supervision they had felt less confident to carry out home-based exercise training at high intensity.

**Conclusion:**

This study highlights the importance of group-based supervised exercise training for patients who engage well in such a setting, and the detrimental impact of disruption to this type of CR service on physical activity levels and exercise capacity. Additionally, learning from the COVID-19 pandemic may inform the development and implementation of remote CR modalities going forward.

## Introduction

The Coronavirus Disease 2019 (COVID-19) pandemic has resulted in extraordinary worldwide public health orders of social distancing and self-isolation. This has led to widespread disruption of centre-based cardiac rehabilitation (CR) programmes and other social opportunities for cardiovascular disease (CVD) patients to engage in heart-healthy exercise. Heart-healthy exercise, in this context, refers to the medically recommended amount and intensity of regular physical activity (PA) to achieve a positive effect in the secondary prevention of CVD. This includes leisure activities and activities of daily living (e.g., walking, hiking, gardening) as well as targeted endurance and strength training (Niebauer et al., 2013; Schwaab et al., 2021).

In Austria, the first COVID-19 lockdown was initiated in mid-March 2020, with closure of all outpatient CR centres and restriction of inpatient rehabilitation to patients with urgent medical indications only (Beiglböck et al., 2020; Desson et al., 2020). During May 2020, restrictions in Austria were gradually lifted, but outpatient CR centres, fitness centres and sports grounds remained closed for group-based exercise and sports activities for a further 2 months. In July 2020, Austrian outpatient CR centres then re-opened under strict hygiene regulations, enabling a gradual return to group-based training for CVD patients.

The COVID-19 pandemic has presented a twofold risk for CVD patients. Firstly, pre-existing cardiovascular co-morbidity exposes CVD patients to high risk of adverse outcome in case of COVID-19 infection (Williamson et al., 2020). The British OpenSAFELY study of over 17,000,000 primary care health records showed a hazard ratio of 1.17 (95% CI = 1.12, 1.22) for COVID-19-related death in those with chronic heart disease (Williamson et al., 2020). CVD patients have therefore been urged to take social distancing measures very seriously. This, however, has in turn led to invariable disruption of CVD patients’ opportunities and established routines for group-based PA (Hall et al., 2021). Exercise-based CR and regular heart-healthy PA are vital measures in the secondary prevention of CVD (Niebauer et al., 2013; Reich et al., 2020). The COVID-19 pandemic has raised a subsequent risk for CVD patients relating to potential deterioration in modifiable CVD risk factors which are amenable to PA (e.g., arterial hypertension, hypercholesterolaemia, obesity, hyperglycaemia), leading to a potential worsening of overall CVD risk status (Chen et al., 2020; Crisafulli and Pagliaro, 2020; Lippi et al., 2020).

We conducted a study to investigate the impact of the COVID-19-related national lockdown and public health restrictions in Austria on CR patients, with respect to maintenance of PA for secondary CVD prevention. The purpose was (1) to assess the impact of the closure of group-based CR training during the first COVID-19 lockdown in spring 2020 on patients’ physical activity, cardiorespiratory fitness, and cardiovascular risk, and (2) to describe the patient experience of lockdown and home-based exercise training during lockdown.

## Materials and Methods

### Study Design

We conducted a mixed methods study with sequential quantitative – qualitative design, in which qualitative data were used to elucidate quantitative data (Schoonenboom and Johnson, 2017; Cresswell and Plano Clark, 2021). The study protocol was reviewed and approved by the Medical Research Ethics Committee of the County of Salzburg (reference 1095/2020) and prospectively registered on ClinicalTrials.gov (identifier NCT04501432).

### Setting and Participants

We recruited participants from a cohort of CR patients who had regularly attended weekly group-based exercise training at the University Institute of Sports Medicine, Prevention and Rehabilitation, Salzburg, Austria, until the COVID-19-related lockdown came into force in mid-March 2020. Patients were in phase IV of CR and had previously completed phase II and/or phase III CR programmes. Phase IV CR describes a patient’s lifelong self-managed heart-healthy lifestyle and includes a recommendation for regular targeted exercise training (Niebauer et al., 2013).

As part of their phase IV CR, patients had been attending weekly group-based training sessions at the study site for several months and up to several years prior to March 2020. These supervised training sessions comprise submaximal cycle ergometer training with 3-lead ECG monitoring followed by resistance training using medical-grade gym equipment. Intensity/resistance of training is regularly revisited and adjusted by staff at the centre, aiming toward medically recommended training targets for CR patients (Niebauer et al., 2013). Patients undergo annual maximal exercise capacity testing as part of their routine care.

At the time of the first COVID-19-related lockdown, the centre was not set up to provide telehealth or telerehabilitation services. During the first weeks of lockdown, staff produced motivational videos with exercise instructions and short training routines for indoor training. Patients were informed by letter that such videos had been produced and were freely accessible to them online.

Participants were recruited from mid-July 2020, when patients were invited to return to train at the centre following lockdown. Eligibility criteria were: pre-lockdown completion of a maximal cycle ergometer test, no new contraindications for maximal exercise testing, no new complaints limiting exercise performance, and no language barrier for qualitative interviews. All participants gave written informed consent to the study.

### Quantitative Assessments

Clinical and socio-demographic data were collected from medical records, including most recent pre-lockdown maximal cycle ergometer test results and submaximal cycle ergometer training session recordings.

The first study visit included post-lockdown medical history and physical exam, anthropometry, resting blood pressure, resting 12-lead electrocardiogram (ECG), and venous blood samples (blood count, electrolytes, glucose, HbA1c, lipids, kidney, and liver enzymes). Overall CVD risk was estimated using the Framingham risk score (FRS) for recurrent cardiovascular events (D’Agostino et al., 2000). Self-reported PA level was obtained using the German short version of the International Physical Activity Questionnaire (IPAQ; Sember et al., 2020). Participants then underwent maximal cycle ergometer testing using the same protocol as pre-lockdown (step test protocol until exhaustion with 12-lead ECG recording) (Balady et al., 2010). Supervising clinicians and patients were unaware of the previous and current performance during ergometry.

Following a resting period of 2–7 days, participants attended the second study visit and completed an endurance training session at their individual pre-lockdown settings. Endurance training was performed on electrically braked cycle ergometers (Ergoline^®^ ergoselect 100, Erlangen, Germany) as high-intensity interval or pyramid training for 40–50 min. The selection between high-intensity interval or pyramid training at the study site is decided by patient preference, as these modalities achieve similar improvements in CR training (Tschentscher et al., 2016). Post-lockdown, all participants completed the same training modality as in their most recent pre-lockdown session. Heart rate was measured continuously with a 3-lead-ECG and monitored by sport scientists.

### Qualitative Interviews

Concluding the second study visit, participants gave audio-recorded semi-structured interviews (Britten, 1995; Pope and Mays, 1995). The interviewer asked pre-defined questions to explore participants’ experiences of the COVID-19 lockdown and the closure of group-based CR training. The interview schedule is provided in Supplementary Table 1.

### Statistical Analysis

Quantitative data were summarised using descriptive statistics. Within-group comparisons of cardiorespiratory fitness and CVD risk status before *versus* after lockdown were conducted using paired *t*-test or Wilcoxon signed rank test (2-tailed, alpha = 0.05). Individual pre-post ergometry results for maximal exercise capacity were categorised (worsened, unchanged, improved) applying a threshold for minimal detectable difference of 13 W (Hellmark and Bäck, 2018). Statistical analyses were conducted using IBM SPSS v26 software.

### Qualitative Analysis

Interview recordings [median (range) duration 44 (21, 96) min; total duration 21:43 h] were transcribed verbatim, anonymised, coded in qualitative analysis software QDA Miner Lite v2.0.8 and interpreted using framework analysis (Gale et al., 2013). Framework categories were defined according to the major topics of the interview schedule, and portions of text were coded to the relevant category. E.g., responses to the question whether participants had been able to maintain their recommended amount of PA during the lockdown were coded to the framework category “Maintaining physical activity and physical fitness.” Within framework categories, more fine-grained codes were then assigned to meaningful units of text. E.g., a respondent’s account of how his wife was encouraging him to keep physically active was labelled “Motivating influence of spouse and other family members,” and so on. The first iteration of coding was prepared by one researcher (IH) and subsequently refined through peer review and further analysis by other study team members (SK, JG, and DW).

### Data Integration

Data integration was conducted using a mixed methods matrix (O’Cathain et al., 2010), in which quantitative and qualitative data for each participant were summarised, allowing comparison of findings within and across participants and identification of patterns in the data. Data integration incorporated Kappa statistics, Fisher’s exact test, and Spearman’s correlation coefficients to corroborate and explain patterns in the data (Bryman, 2006).

## Results

**FIGURE 1:**
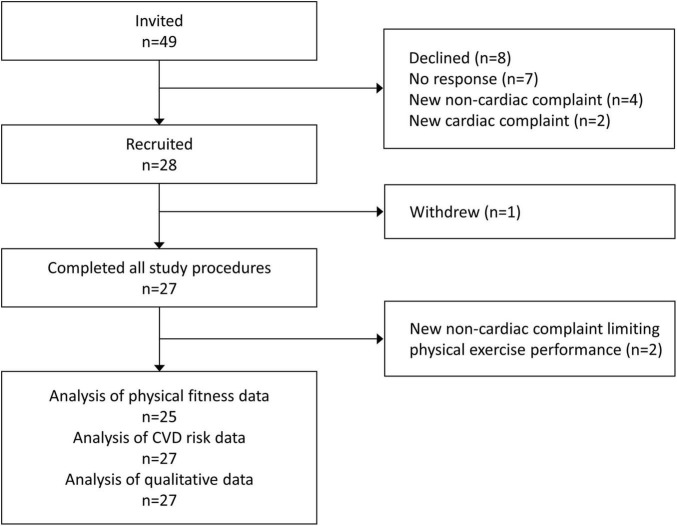
Study flow diagram.

Group-based exercise training at the outpatient CR centre was suspended from 16th March until 17th July 2020. From mid-July until the end of October 2020, we recruited 28 (57%) of 49 eligible patients. One withdrew, and 27 completed all study procedures (Figure 1). Participant characteristics are presented in Table 1.

**TABLE 1:** Participant characteristics.

Characteristic	Categories	*N* = 27
Age, years (mean, SD, range)		69 (7, 55–82)
Sex	Female	6 (22%)
	Male	21 (78%)
Marital status	Married/civil partnership	24 (89%)
	Single	3 (11%)
Employment status	Retired	18 (67%)
	Employed/self-employed	8 (30%)
	Homemaker	1 (4%)
Residence	Urban	15 (44%)
	Rural	12 (56%)
Primary indication for cardiac rehabilitation (ICD-10 codes)	Ischaemic heart diseases (I20-I25)	21 (78%)
	Heart failure (I50)	2 (8%)
	Pulmonary heart disease and diseases of pulmonary circulation (I26-I28)	1 (4%)
	Non-rheumatic aortic valve disorders (I35)	1 (4%)
	Myocarditis (I41)	1 (4%)
	Malformation of coronary vessels (Q24.5)	1 (4%)
Time since first cardiac event, years (median, IQR)^a^		8 (5.5–9)
Diabetes mellitus	Yes	2 (7%)
	No	25 (93%)
Smoking status	Non-smoker	18 (67%)
	Ex-smoker	9 (33%)
	Current smoker	–
IPAQ physical activity level^a^	Low	5 (18%)
	Moderate	6 (22%)
	High	13 (48%)
	Missing	3 (11%)

*Data are n (%) unless stated otherwise. ICD-10, International Classification of Diseases, Tenth Revision; IPAQ, International Physical Activity Questionnaire.*

*^a^At time of data collection.*

### Exercise Capacity and Cardiovascular Risk

**FIGURE 2:**
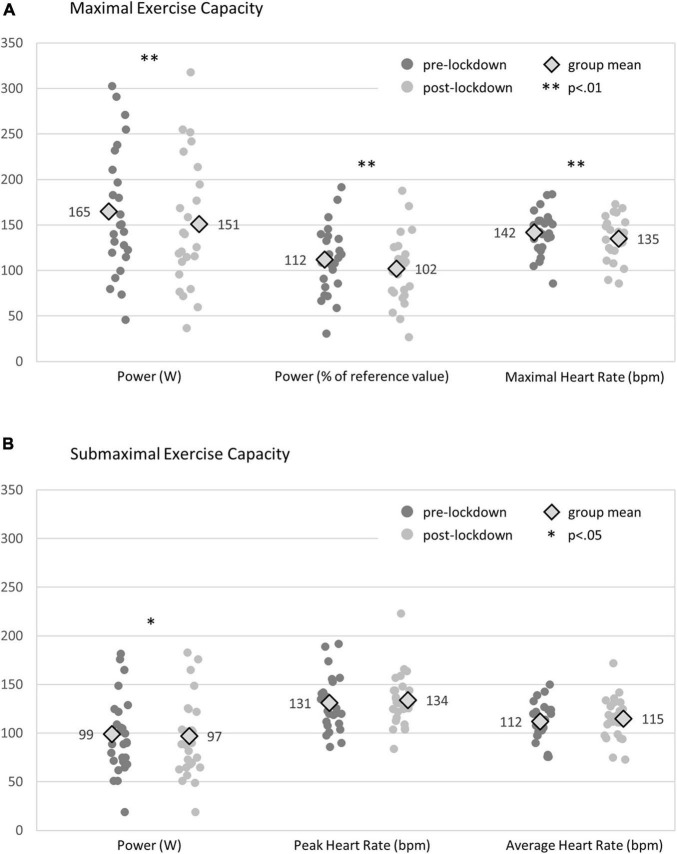
Change in maximal **(A)** and submaximal **(B)** exercise capacity.

Two participants were excluded from analysis of cardiorespiratory fitness data, due to subsequent diagnosis of new non-cardiac complaints limiting exercise performance. The median (IQR) time period between participants’ most recent pre-lockdown maximal cycle ergometer tests and the beginning of lockdown was 6 (5, 9) months. The median (IQR) time period between pre- and post-lockdown maximal cycle ergometer tests was 11 (10, 20) months. The median (IQR) time period between pre-lockdown submaximal cycle ergometer training session and the beginning of lockdown was 1 (1, 2) week. The median (IQR) time period between pre- and post-lockdown submaximal cycle ergometer training sessions was 5 (5, 7) months. With regard to individual exercise capacity, 14 (56%) participants had worsened, 10 (40%) were unchanged, and 1 (4%) had improved post-lockdown. FRS for recurrent cardiovascular events had worsened in 7 (26%), remained unchanged in 12 (44%), and improved in 8 (30%) participants. At group level, maximal and submaximal exercise capacity had significantly reduced, whereas CVD risk factors remained unchanged from pre- to post-lockdown (Table 2 and Figure 2).

**TABLE 2:** Cardiorespiratory fitness and cardiovascular disease risk status pre- and post-lockdown.

Outcome	Parameter	Pre	Post	Difference	*P*-value
Maximal cycle ergometry^a^	Power (W)	165 (70)	151 (70)	−14 (12)	<0.001
	Power (% of reference value)	112 (37)	102 (38)	−10 (10)	<0.001
	Maximal heart rate (bpm)	142 (24)	135 (24)	−7 (9)	0.003
Submaximal cycle ergometer training session^b^	Power (W)	99 (40)	97 (40)	−2 (5)	0.038
	Peak heart rate (bpm)	131 (28)	134 (28)	3 (22)	0.73
	Average heart rate (bpm)	112 (19)	115 (21)	3 (11)	0.30
CVD risk status	Resting systolic blood pressure (mmHg)	121 (20)	124 (18)	3 (20)	0.46
	Weight (kg)	82.5 (25.2)	82.4 (15.6)	−0.1 (3.4)	0.87
	Body mass index (kg/m^2^)	27.13 (4.8)	27.12 (4.8)	−0.01 (1.0)	0.94
	Cholesterol (mg/dl)	169 (53)	171 (55)	2 (28)	0.74
	Triglycerides (mg/dl)	137 (70)	145 (90)	8 (56)	0.45
	HDL cholesterol (mg/dl)	59 (14)	65 (16)	6 (7)	<0.001
	LDL cholesterol (mg/dl)	88 (49)	81 (50)	−7 (23)	0.11
	Glucose (mg/dl)^c^	102 (18)	96 (10)	−6 (17)	0.11
	HbA1c (%)^d^	6.0 (0.3)	6.0 (0.2)	<0.1	0.12
	CVD risk (%)^e^	7.0 (2.8)	6.9 (2.4)	−0.1 (0.9)	0.61

*Data are arithmetic mean (SD). P-values by paired t-test or Wilcoxon signed rank test (2-tailed, alpha = 0.05). CVD, cardiovascular disease; HDL, high density lipoprotein; LDL, low density lipoprotein.*

*^a^n = 25; median (IQR) time period between pre- and post-lockdown test = 11 (10, 20) months.*

*^b^Median (IQR) time period between pre- and post-lockdown training session = 5 (5, 7) months.*

*^c^n = 25.*

*^d^n = 23.*

*^e^Framingham risk score for recurrent cardiovascular event within 2 years.*

### Patient Experience During Lockdown

Reflecting on their ability to maintain PA and cardiorespiratory fitness since the beginning of the COVID-19-related lockdown, almost all participants (*n* = 25) stated that they had found alternatives to keep physically active. Activities included self-directed training at home or outdoors (home trainer, jogging, cycling), going for walks and hikes, and household activities such as gardening and chopping firewood. Despite this, 17 (63%) said they had not been able to maintain the amount and intensity of exercise (C09, Table 3) and 15 (56%) felt their fitness had worsened (C23, Table 3).

**TABLE 3:** Anonymised quotes from qualitative interviews.

Speaker	Quote	Code	Framework category
C01	“Well, with regard to running, it (physical fitness) has definitely improved. I know that. Because I monitor practically all those parameters, distance average pulse, speed, and so on. So, there you can–, with regard to those parameters, I have improved.”	Improved exercise levels and fitness	Maintaining physical activity and physical fitness
C05	“I just said, I have to walk a little bit, I just have to, if you can’t go to rehab any more, just go marching.”	Intrinsic motivation to exercise during lockdown	Maintaining physical activity and physical fitness
C07	“I have an old mobile. I don’t have a smartphone. And that is also too large. So, for me that is too cumbersome. Because the small mobile I slide in my pocket and done. Smartphones are definitely an amazing thing. But I see that certain people, they are almost addicted to it. And I don’t understand that at all.”	Reasons for non-use	Digital technology for self-directed physical activity
C08	“I mean, it’s just so silly. You could just as well do something on your own. But that’s just not fun, isn’t it? Then you come here, and it just flows automatically. (…) But alone, it’s no fun.”	Missing the motivation from training with others	Closure of group-based cardiac rehabilitation classes
C09	“And so, unfortunately, that (exercise programme) then successively reduced. And then with the Corona period and all the excuses one comes up with (…) Idleness, neglect. Yes, the ‘inner devil’ gets stronger.”	Unable to maintain exercise levels	Maintaining physical activity and physical fitness
C11	“No, I always took care that I had generous amounts of medication at home. Because you don’t know, is there a shortage or something. No, that worked well. It also worked well with our family doctor. No problem.”	Obtaining prescription medication during lockdown	Medication adherence, smoking, and diet
C12	“So, I have this movement app, I got that. But you realise, you say: Now I did, I’m still missing a few steps to reach the target. So, I’ll walk another small round.”	Step-counter	Digital technology for self-directed physical activity
C13	“There was nobody who said, and now you do this and then you do this. Except for my wife: ‘And now you do this’.”	Motivating influence of spouse and other family members	Maintaining physical activity and physical fitness
C15	“Funnily enough, for the first time in my life, I became really lazy. Which then also had its consequences.”	Unable to maintain exercise levels	Maintaining physical activity and physical fitness
C16	“Because there is not enough time for cooking when I’m at work. (…) That just isn’t healthy food. So, regarding my diet, I tend to try that, in my free time when I’m off work, I eat more consciously. (…) But definitely, during lockdown I was able to make use of that. To eat more healthily.”	Opportunities to improve diet during lockdown	Medication adherence, smoking, and diet
C17	“I missed that, the organised (group exercise). Because on your own – I don’t know what you are like, but it is difficult on your own. Of course, if you have a partner who is active and says, ‘now, let’s do something,’ then – But if you see that they are not able to participate, you do get idle.”	De-motivating influence of spouse not engaging in exercise	Maintaining physical activity and physical fitness
C18	“And my guilty pleasure is sweets and carbohydrates. Bread and so on. And this was difficult during lockdown. Because you could see that I tended to reward myself with food. And also, because we spent a lot of time at home, I did change my eating habits.”	Difficulties maintaining heart-healthy diet during lockdown	Medication adherence, smoking, and diet
C22	“The training. Here. At the Sports Medicine (CR centre). Because that was something like a ‘jour fixe.’ Once a week, you come here, you do your training, you see how you are doing. You have the readings in front of you and that. And I actually missed that.”	Regretting the lack of a weekly fixture for group exercise	Closure of group-based cardiac rehabilitation classes
C23	“But I did think, I wouldn’t say that I made any improvements in my fitness during lockdown. That would be self-deception. (…) And because of this, I would say that in my estimation I am about ten, fifteen to twenty percent weaker compared to before the lockdown. Gut feeling.”	Worsening physical fitness pre *versus* post lockdown	Maintaining physical activity and physical fitness
C24	“For me, that’s the enemy. The computer, that’s the enemy for me. Because I never learned to touch-type, because I was in construction. And then I had to work with the computer, enter all the cables and things. (…) Yes, I actually don’t need it.”	Reasons for non-use	Digital technology for self-directed physical activity
C25	“…you come here and do your training, you are monitored. You just have a good feeling, when you leave. You say, okay, they did have a look at that, and if there would have been anything, they would have reacted and would have told me.”	Confidence through being monitored during exercise training	Closure of group-based cardiac rehabilitation classes

With regard to other secondary prevention behaviour, participants reported that lockdown had not affected medication adherence or (non)smoking (C11, Table 3). Most participants generally observed heart-healthy dietary recommendations and had continued to do so during lockdown. Some participants said they had eaten more unhealthy food during lockdown and gained weight (C18, Table 3). Conversely, others found that lockdown created more time for home-cooking, causing them to reflect on and improve their diet (C16, Table 3). In general, participants regretted the lack of a weekly “fixture” for exercise (C22, Table 3). Many missed the sense of community at the outpatient rehabilitation centre and the motivation from training together with others (C08, Table 3). Several participants stated that without professional supervision they felt less confident or unsafe to train at the same (high) intensity as at the rehabilitation centre (C25, Table 3). Many participants also commented that they had not been able to find good alternatives for the strength training component for which medical-grade weightlifting equipment was available at the centre. Post-lockdown, 22 (82%) participants continued with regular weekly training sessions at the centre, while 5 (18%) decided not to (e.g., ongoing fear of contracting COVID-19).

### Findings From Data Integration

In the integration of quantitative and qualitative data, self-estimated levels of cardiorespiratory fitness from qualitative interviews (worsened, unchanged, improved) matched findings from maximal ergometry in 18 (67%) participants, showing fair agreement (Kappa 0.36, *p* = 0.04). Only 3 (11%) participants overestimated their cardiorespiratory fitness (Kappa 0.66, *p* < 0.001). This indicates good judgement and supports the credibility of patients’ reflections on PA during lockdown.

Self-reported ability to maintain heart-healthy PA was not associated with age, sex, place of residence, or employment status (Table 4), placing emphasis on qualitative data to explain motivations and barriers for PA. With respect to employment status, it is noteworthy that for most participants the COVID-19 pandemic had not caused any financial insecurity. Only one participant had demonstrably improved his cardiorespiratory fitness over the lockdown period. His work-related travel had been reduced and meetings were conducted remotely, creating more time for self-directed individual exercise. This participant was familiar with heart rate and activity monitoring via smartwatch and confident in pushing himself in self-directed exercise (C01, Table 3). In contrast, the other participants described various experiences, from those who kept intrinsically motivated to exercise (C05, Table 3) to those who struggled (C15, Table 3). A prominent facilitator/barrier for PA for many participants was the motivating influence of spouses or other family members who encouraged or joined in PA (C13, Table 3), and – conversely – the demotivating influence of next of kin not willing or unable to engage in PA (C17, Table 3).

**TABLE 4:** Associations with ability to maintain physical activity and Framingham risk score.

Variables		Association^a^
Ability to maintain physical activity (self-report)	Age	−0.237 (*p* = 0.233)
	Sex	*p* = 0.608 (Fisher’s exact)
	Place of residence (urban *versus* rural)	*p* > 0.999 (Fisher’s exact
	Employment status (working *versus* retired)	*p* = 0.169 (Fisher’s exact)
Δ Framingham risk score^b,c^	Δ Total cholesterol^b^	−0.515 (*p* = 0.006)
	Δ HDL cholesterol^b^	0.543 (*p* = 0.003)
	Δ LDL cholesterol^b^	−0.343 (*p* = 0.079)
	Δ Body weight^b^	−0.010 (*p* = 0.960)
	Δ Body mass index^b^	−0.007 (*p* = 0.973)
	Ability to maintain physical activity (self-report)	−0.134 (*p* = −0.507)
	IPAQ physical activity level	0.114 (*p* = 0.570)
	Maximal exercise capacity post-lockdown (% of the reference value)	0.290 (*p* = 0.143)
	Δ Maximal exercise capacity^b^	−0.140 (*p* = 0.485)

*HDL, high-density lipoprotein; IPAQ, International Physical Activity Questionnaire; LDL, low-density lipoprotein.*

*^a^Spearman’s Rho unless stated otherwise.*

*^b^Δ refers to change from pre- to post-lockdown.*

*^c^Framingham risk score for recurrent cardiovascular event within 2 years.*

The use of digital technologies to support or enhance self-directed PA during lockdown in this cohort was limited. Only three participants described the deliberate use of wearables and fitness apps (C01, Table 3), some reported using the step counter function of their smartphone or fitness tracker (C12, Table 3), and some had accessed digital platforms for information on exercise, including online videos created by staff at the CR centre specifically for patients in COVID-19 quarantine. Many participants were either disinterested or sceptical regarding the usefulness of digital technologies for PA. Reasons included doubt in the accuracy of wearable measurements, discomfort of carrying/wearing a device, psychological pressure and anxiety created by self-tracking, and low affinity toward or deliberate rejection of digital technologies (C07, C24, Table 3). There were no distinct patterns when these qualitative accounts were compared with quantitative PA and cardiorespiratory fitness data.

Individual changes in FRS were small (0.5–3% points). These changes were associated with changes in blood lipid levels from pre- to post-lockdown, but not with other parameters (Table 4). Cholesterol lowering medication had remained unchanged, except for one patient who had reduced, and one who had increased his statin dose. These two patients’ FRS post-lockdown had worsened and improved, respectively. Participants’ qualitative accounts of their dietary habits during lockdown did not offer any explanatory patterns with regard to changes in cholesterol or FRS.

## Discussion

In this cohort of CR patients, cardiovascular risk factors had remained unchanged after lockdown, but maximal exercise capacity had significantly reduced by 8.5%. Despite self-directed physical activities during lockdown, patients were unable to maintain exercise levels equivalent to regular group-based CR training. To put these findings into context, 6–12 months of weekly outpatient CR training in this setting typically achieve a 10% increase in maximal exercise capacity (Reich et al., 2020). Considering the average time period of 6 months between pre-lockdown maximal exercise capacity tests and the beginning of lockdown, it is therefore possible that the negative effect of lockdown on maximal exercise capacity may have been underestimated in this analysis.

For the majority of patients, pre-lockdown data for submaximal exercise capacity were available from within 1 to 2 weeks of the beginning of lockdown. These data showed a statistically significant reduction by 2% following lockdown, which corroborates the trend seen in maximal exercise capacity and further supports the temporal aspect of the impact of lockdown.

We emphasise that our findings do not speak to a comparison of centre-based versus home-based CR programmes, for which there is high-level evidence of similar effectiveness (Anderson et al., 2017). Rather, our findings illustrate the impact of the disruption to weekly group-based supervised exercise training during the COVID-19-related lockdown, which forced patients to find their own home-based alternatives.

### Population-Level Physical Activity During the COVID-19 Pandemic

Key findings from this study are in line with other research on PA behaviour during the COVID-19 pandemic. Already early on in the COVID-19 pandemic, concerns were raised about potential negative consequences of social isolation on preventive health behaviour and subsequent worsening of non-communicable chronic diseases (Lippi et al., 2020). One year later, a systematic review (66 studies, >86,000 individuals) demonstrated that overall levels of PA in healthy adolescents and adults had declined and sedentary behaviour had increased (Stockwell et al., 2021). A rapid review (26 studies, >23,000 respondents) showed that PA had also decreased in most patient groups with disabilities and chronic conditions (de Boer et al., 2021). Moreover, an online survey of over 11,000 adults in 11 countries across Europe, Asia, and North and South America indicated that more stringent national COVID-19 restrictions were associated with greater likelihood of insufficient PA (adjusted OR = 1.22, 95% CI = 1.03, 1.45) (Ding et al., 2021).

This evidence derives mainly from self-reported PA data in healthy and non-cardiac patient populations, but some studies also provide objectively tracked data from cardiac patient groups, e.g., demonstrating over 25% reduction in activity data from patients with implantable cardioverter-defibrillators (Malanchini et al., 2020) and a 16% decrease in step count of heart failure patients (Vetrovsky et al., 2020). This research describes a distinct global trend of reduced PA due to COVID-19 public health restrictions, which is mirrored by findings from our study. In contrast, reports of changes in dietary habits present mixed patterns, including reduced alcohol consumption and healthier eating habits during lockdown due to less eating out and more home cooking (Ammar et al., 2020; Rossinot et al., 2020; Flanagan et al., 2021). This was also reflected in our study findings, with participants describing positive as well as negative changes to their eating habits during lockdown.

### Determinants of Physical Activity During the COVID-19 Pandemic

In a survey of 1,809 adults in the United States, the most common reason for increasing PA during the COVID-19 pandemic was more time, whereas the most common barriers for PA were motivation and worry/stress (Knell et al., 2020). A survey of 427 adults in Belgium showed that worsened time allocation, experiencing PA as effortful, and lack of family encouragement were significant barriers to PA, whereas better time allocation and not experiencing PA as effortful predicted higher levels of PA (Symons et al., 2021). Another survey of 1,521 adults in the United Kingdom showed that physical opportunity and reflective motivation were the most consistent predictors of PA behaviour, while automatic motivation was not or negatively associated with PA (Spence et al., 2021). This indicates that in the radically different context of the COVID-19 pandemic, reflective motivational processes (e.g., making plans, evaluating what has happened) appear more relevant than automatic processes that usually influence habitual PA behaviour during our daily routines. Specific to CVD, a longitudinal survey of 1,565 patients during the first COVID-19 lockdown in the Netherlands indicated that the amount of moderate to vigorous PA was lower in women, patients with heart failure, those with limited possibilities for PA, and those who were fearful of COVID-19 infection (van Bakel et al., 2021).

These findings from quantitative observational studies match qualitative data from our study, in particular with respect to participants’ reflective motivation, family encouragement, and time. Anecdotally, several of the patients who declined participation in our study indicated that this was due fear of COVID-19 infection. Other qualitative research has also provided in-depth and differentiated accounts of individuals’ experiences during lockdown which align with our findings (Petersen et al., 2021; Schaffler et al., 2021), drawing out both positive and negative consequences of circumstances during lockdown and emphasising the need to offer targeted and contextually relevant strategies for supporting PA during the COVID-19 pandemic.

### Supporting Physical Activity During the COVID-19 Pandemic

Early recognition of the detrimental impact of COVID-19 social isolation measures on PA behaviour led to a swift response in promoting home-based training through prominent public health campaigns, including guidance for home-based exercise online and via digital platforms (Dwyer et al., 2020; Nyenhuis et al., 2020). There was a demonstrable peak in online searches for “exercise” during the first phase of the pandemic in Australia, the United Kingdom, and the United States, indicating increased public interest in home-based PA (Ding et al., 2020). Use of PA apps and various digital platforms correlated with maintenance of PA and meeting PA recommendations in healthy adults and adolescents in Australia and the United States (Yang and Koenigstorfer, 2020; Parker et al., 2021). However, only a proportion of the population are accessing apps and digital platforms for PA (e.g., 39.5% in Australia) (Parker et al., 2021), and use of these types of digital support as captured in observational surveys may indicate individuals with *a priori* higher health literacy and greater motivation for PA, rather than representing a cause of increased PA behaviour. Digital interventions for increasing PA have also been shown to be more efficacious for individuals with high (as opposed to low) socio-economic status (Western et al., 2021). Moreover, these digital offers are typically aimed at the general/healthy population, while more specific and tailored solutions are required to support clinical populations such as patients with CVD.

Recommendations for supporting PA during the COVID-19 pandemic suggest that vulnerable groups, such as the elderly and patients, should be offered supervised home-based exercise programmes (Bentlage et al., 2020; Neubeck et al., 2020). Authors highlight the benefits of PA on mental health and the importance of supporting mental as well as physical health (Bentlage et al., 2020; Neubeck et al., 2020). The application of wearable tracking devices, providing an overview of daily activities to help reduce inactivity and improve cardiovascular parameters, is a prominent recommendation in the literature. Importantly, there needs to be consideration of access to digital technologies, e.g., access to the internet and affordability of devices, and individuals need to be offered support and education in applying these technologies (Bentlage et al., 2020; Neubeck et al., 2020). In addition, it is recommended that digital support programmes include explanations of the importance of PA, setting of realistic exercise goals according to the individual’s health status, and integration of family members for added motivational impact (Bentlage et al., 2020).

### Future Directions

With regard to delivering health care services for CVD patients, the role of telehealth systems and digital technology for the continuation of routine cardiac care and rehabilitation during the COVID-19 pandemic has been emphasised (Neubeck et al., 2020; Scherrenberg et al., 2020). This raises organisational implications, including preparedness of health care providers in terms of availability of equipment, workforce skills and management of workflows, and financial implications regarding reimbursement mechanisms for remote health care delivery (Neubeck et al., 2020; Scherrenberg et al., 2020). Of note, telehealth encompasses not only sophisticated digital solutions but also commonplace communication such as telephone calls, text messaging and email, which may already offer successful strategies (Thomas et al., 2020).

In the long term, it is anticipated that the COVID-19 pandemic will act as a catalyst for telehealth, telerehabilitation, and digital technologies, enabling more consistent incorporation of remote delivery modes for CR and rehabilitation in other clinical specialities (Scherrenberg et al., 2020; Laoutaris et al., 2021). There is high-quality evidence for the efficacy of telehealth interventions within CR settings (Gruska et al., 2020; Thomas et al., 2020; Schwaab et al., 2021), which should reassure providers that remote CR modalities may be offered safely to supplement centre-based CR according to current evidence-based clinical guidelines (Gruska et al., 2020; Schwaab et al., 2021).

This study emphasises the need for political, technical and organisational preparedness to offer remote support for PA via digital and non-digital strategies, taking into account individual needs, preferences, health literacy and digital literacy of CVD patients. There is an opportunity to build on insights gained during the COVID-19 pandemic to develop and implement remote rehabilitation modalities going forward.

### Limitations

This study was observational and opportunistic, responding to sudden and exceptional circumstances created by the global COVID-19 pandemic. To address our research questions, we relied on retrospective medical record data in addition to prospectively collected data. Cardiopulmonary exercise testing (CPET), as opposed to maximal exercise capacity testing, would have been a preferable measure of cardiorespiratory fitness for our investigation; however, CPET is not conducted routinely for CR patients at the study site, and therefore CPET data were not available for the pre-lockdown timepoint. The time period between participants’ pre-lockdown maximal exercise capacity tests and the beginning of lockdown presents an additional limitation.

We acknowledge that our assessment of PA was limited due to the lack of an objective measure such as accelerometry, and that we did not capture quantitative dietary information. We acknowledge the potential for selection bias, whereby study recruits might represent more exercise-conscious patients who were more motivated to return to group-based CR sessions after lockdown. Participants’ mental health in association with PA during lockdown was not explicitly addressed in our study, although it is an important aspect described in the literature (Stockwell et al., 2021).

## Conclusion

This study adds to the currently limited literature on the impact of COVID-19-related social isolation on patients with CVD. The findings highlight the importance of providing group-based opportunities for supervised high-intensity training to patients who engage well in such a setting, and the detrimental impact of disruption to this type of CR service on PA levels and exercise capacity.

## Data Availability Statement

The raw data supporting the conclusions of this article will be made available by the authors, without undue reservation.

## Ethics Statement

The studies involving human participants were reviewed and approved by the Medical Research Ethics Committee of the County of Salzburg. The patients/participants provided their written informed consent to participate in this study.

## Author Contributions

SK, MS, and JN conceived and designed the study. MS, SD, AE, BM, and BR collected quantitative data. SK, JG, IH, and DW collected qualitative data. SK, MS, and IH conducted data analyses with contributions from all authors to interpretation and analysis. SK drafted the manuscript. All authors reviewed and approved the manuscript for publication.

## Conflict of Interest

The authors declare that the research was conducted in the absence of any commercial or financial relationships that could be construed as a potential conflict of interest.

## Publisher’s Note

All claims expressed in this article are solely those of the authors and do not necessarily represent those of their affiliated organizations, or those of the publisher, the editors and the reviewers. Any product that may be evaluated in this article, or claim that may be made by its manufacturer, is not guaranteed or endorsed by the publisher.

## Abbreviations

COVID-19: Coronavirus Disease 2019
CPET: Cardiopulmonary Exercise Testing
CR: Cardiac Rehabilitation
CVD: Cardiovascular Disease
ECG: Electrocardiogram
FRS: Framingham Risk Score
IPAQ: International Physical Activity Questionnaire
PA: Physical Activity.

## References

[B1] Ammar A. Brach M. Trabelsi K. Chtourou H. Boukhris O. Masmoudi L. (2020). Effects of COVID-19 home confinement on eating behaviour and physical activity: results of the ECLB-COVID19 international online survey. *Nutrients* 12:E1583. 10.3390/nu12061583 32481594 PMC7352706

[B2] Anderson L. Sharp G. A. Norton R. J. Dalal H. Dean S. G. Jolly K. (2017). Home-based versus centre-based cardiac rehabilitation. *Cochrane Database Syst. Rev.* 6:CD0 07130. 10.1002/14651858.CD007130.pub4 PMC6481471 28665511

[B3] Balady G. J. Arena R. Sietsema K. Myers J. Coke L. Fletcher G. F. (2010). Clinician’s Guide to cardiopulmonary exercise testing in adults: a scientific statement from the American Heart Association. *Circulation* 122 191–225. 10.1161/CIR.0b013e3181e52e69 20585013

[B4] Beiglböck M. Grohs P. Hermisson J. Nordborg M. Schachmayer W. (2020). *Stellungnahme zur COVID19 Krise, 30.03.2020. Executive Summary.* Available online at: https://www.amz-gmbh.net/breaking-news-stellungnahme-zur-covid19-krise-30-3-2019/ (accessed January 18, 2022).

[B5] Bentlage E. Ammar A. How D. Ahmed M. Trabelsi K. Chtourou H. (2020). Practical recommendations for maintaining active lifestyle during the COVID-19 pandemic: a systematic literature review. *Int. J. Environ. Res. Public Health* 17:E6265. 10.3390/ijerph17176265 32872154 PMC7503956

[B6] Britten N. (1995). Qualitative interviews in medical research. *BMJ* 311 251–253. 7627048 10.1136/bmj.311.6999.251 PMC2550292

[B7] Bryman A. (2006). Integrating quantitative and qualitative research: how is it done? *Qual. Res.* 6 97–113. 10.1177/1468794106058877

[B8] Chen P. Mao L. Nassis G. P. Harmer P. Ainsworth B. E. Li F. (2020). Coronavirus disease (COVID-19): the need to maintain regular physical activity while taking precautions. *J. Sport Health Sci.* 9 103–104. 10.1016/j.jshs.2020.02.001 32099716 PMC7031771

[B9] Cresswell J. Plano Clark V. (2021). *Designing and Conducting Mixed Methods Research*, 3rd Edn. London: SAGE.

[B10] Crisafulli A. Pagliaro P. (2020). Physical activity/inactivity and COVID-19. *Eur. J. Prev. Cardiol.* 28, e24–e26. 10.1177/2047487320927597 PMC7928968 33611457

[B11] D’Agostino R. B. Russell M. W. Huse D. M. Ellison R. C. Silbershatz H. Wilson P. W. (2000). Primary and subsequent coronary risk appraisal: new results from the Framingham study. *Am. Heart J.* 139 272–281. 10.1067/mhj.2000.96469 10650300

[B12] de Boer D. R. Hoekstra F. Huetink K. I. M. Hoekstra T. Krops L. A. Hettinga F. J. (2021). Physical activity, sedentary behavior and well-being of adults with physical disabilities and/or chronic diseases during the first wave of the COVID-19 pandemic: a rapid review. *Int. J. Environ. Res. Public Health* 18:6342. 10.3390/ijerph18126342 34208156 PMC8296179

[B13] Desson Z. Lambertz L. Peters J. W. Falkenbach M. Kauer L. (2020). Europe’s Covid-19 outliers: German, Austrian and Swiss policy responses during the early stages of the 2020 pandemic. *Health Policy Technol.* 9 405–418. 10.1016/j.hlpt.2020.09.003 33520639 PMC7834269

[B14] Ding D. Del Pozo, Cruz B. Green M. A. Bauman A. E. (2020). Is the COVID-19 lockdown nudging people to be more active: a big data analysis. *Br. J. Sports Med.* 54 1183–1184. 10.1136/bjsports-2020-102575 32605932

[B15] Ding K. Yang J. Chin M.-K. Sullivan L. Durstine J. L. Violant-Holz V. (2021). Physical activity among adults residing in 11 countries during the COVID-19 pandemic lockdown. *Int. J. Environ. Res. Public Health* 18:7056. 10.3390/ijerph18137056 34280992 PMC8297220

[B16] Dwyer M. J. Pasini M. De Dominicis S. Righi E. (2020). Physical activity: benefits and challenges during the COVID-19 pandemic. *Scand. J. Med. Sci. Sports* 30 1291–1294. 10.1111/sms.13710 32542719 PMC7323175

[B17] Flanagan E. W. Beyl R. A. Fearnbach S. N. Altazan A. D. Martin C. K. Redman L. M. (2021). The impact of COVID-19 stay-at-home orders on health behaviors in adults. *Obesity* 29 438–445. 10.1002/oby.23066 33043562 PMC7675243

[B18] Gale N. K. Heath G. Cameron E. Rashid S. Redwood S. (2013). Using the framework method for the analysis of qualitative data in multi-disciplinary health research. *BMC Med. Res. Methodol.* 13:117. 10.1186/1471-2288-13-117 24047204 PMC3848812

[B19] Gruska M. Aigner G. Altenberger J. Burkart-Küttner D. Fiedler L. Gwechenberger M. (2020). Recommendations on the utilization of telemedicine in cardiology. *Wien Klin Wochenschr* 132 782–800. 10.1007/s00508-020-01762-2 33259003

[B20] Hall G. Laddu D. R. Phillips S. A. Lavie C. J. Arena R. (2021). A tale of two pandemics: how will COVID-19 and global trends in physical inactivity and sedentary behavior affect one another? *Prog. Cardiovasc. Dis.* 64 108–110. 10.1016/j.pcad.2020.04.005 32277997 PMC7194897

[B21] Hellmark M. Bäck M. (2018). Test-retest reliability and responsiveness to change of clinical tests of physical fitness in patients with acute coronary syndrome included in the SWEDEHEART register. *Eur. J. Cardiovasc. Nurs.* 17 486–495. 10.1177/1474515117743978 29192797

[B22] Knell G. Robertson M. C. Dooley E. E. Burford K. Mendez K. S. (2020). Health behavior changes during COVID-19 pandemic and subsequent “stay-at-home” orders. *Int. J. Environ. Res. Public Health* 17:E6268. 10.3390/ijerph17176268 32872179 PMC7504386

[B23] Laoutaris I. D. Dritsas A. Adamopoulos S. (2021). Cardiovascular rehabilitation in the COVID-19 era: “a phoenix arising from the ashes? *Eur. J. Prev. Cardiol.* Online ahead of print. 10.1093/eurjpc/zwab116 34179989 PMC8344487

[B24] Lippi G. Henry B. M. Sanchis-Gomar F. (2020). Physical inactivity and cardiovascular disease at the time of coronavirus disease 2019 (COVID-19). *Eur. J. Prev. Cardiol.* 27 906–908. 10.1177/2047487320916823 32270698 PMC7717305

[B25] Malanchini G. Malacrida M. Ferrari P. Leidi C. Ferrari G. Racheli M. (2020). Impact of the Coronavirus disease-19 outbreak on physical activity of patients with implantable cardioverter defibrillators. *J. Card. Fail.* 26 898–899. 10.1016/j.cardfail.2020.08.005 32827643 PMC7438266

[B26] Neubeck L. Hansen T. Jaarsma T. Klompstra L. Gallagher R. (2020). Delivering healthcare remotely to cardiovascular patients during COVID-19?: a rapid review of the evidence. *Eur. J. Cardiovasc. Nurs.* 19 486–494. 10.1177/1474515120924530 32380858 PMC7717235

[B27] Niebauer J. Mayr K. Tschentscher M. Pokan R. Benzer W. (2013). Outpatient cardiac rehabilitation: the Austrian model. *Eur. J. Prev. Cardiol.* 20 468–479. 10.1177/2047487312446137 22508693

[B28] Nyenhuis S. M. Greiwe J. Zeiger J. S. Nanda A. Cooke A. (2020). Exercise and fitness in the age of social distancing during the COVID-19 pandemic. *J. Allergy Clin. Immunol. Pract.* 8 2152–2155. 10.1016/j.jaip.2020.04.039 32360185 PMC7187829

[B29] O’Cathain A. Murphy E. Nicholl J. (2010). Three techniques for integrating data in mixed methods studies. *BMJ* 341:c4587. 10.1136/bmj.c4587 20851841

[B30] Parker K. Uddin R. Ridgers N. D. Brown H. Veitch J. Salmon J. (2021). The use of digital platforms for adults’ and adolescents’ physical activity during the COVID-19 pandemic (Our Life at Home): survey study. *J. Med. Internet Res.* 23:e23389. 10.2196/23389 33481759 PMC7857525

[B31] Petersen J. A. Naish C. Ghoneim D. Cabaj J. L. Doyle-Baker P. K. McCormack G. R. (2021). Impact of the COVID-19 pandemic on physical activity and sedentary behaviour: a qualitative study in a Canadian city. *Int. J. Environ. Res. Public Health* 18:4441. 10.3390/ijerph18094441 33922094 PMC8122654

[B32] Pope C. Mays N. (1995). Reaching the parts other methods cannot reach: an introduction to qualitative methods in health and health services research. *BMJ* 311 42–45. 10.1136/bmj.311.6996.42 7613329 PMC2550091

[B33] Reich B. Benzer W. Harpf H. Hofmann P. Mayr K. Ocenasek H. (2020). Efficacy of extended, comprehensive outpatient cardiac rehabilitation on cardiovascular risk factors: a nationwide registry. *Eur. J. Prev. Cardiol.* 27 1026–1033. 10.1177/2047487319898958 31937125

[B34] Rossinot H. Fantin R. Venne J. (2020). Behavioral changes during COVID-19 confinement in France: a web-Based Study. *Int. J. Environ. Res. Public Health* 17:E8444. 10.3390/ijerph17228444 33202686 PMC7696923

[B35] Schaffler Y. Gächter A. Dale R. Jesser A. Probst T. Pieh C. (2021). Concerns and support after one year of COVID-19 in Austria: a qualitative study using content analysis with 1505 participants. *Int. J. Environ. Res. Public Health* 18:8218. 10.3390/ijerph18158218 34360512 PMC8346103

[B36] Scherrenberg M. Wilhelm M. Hansen D. Völler H. Cornelissen V. Frederix I. (2020). The future is now: a call for action for cardiac telerehabilitation in the COVID-19 pandemic from the secondary prevention and rehabilitation section of the European Association of Preventive Cardiology. *Eur. J. Prev. Cardiol.* Online ahead of print. 10.1177/2047487320939671 PMC7928994 32615796

[B37] Schoonenboom J. Johnson R. B. (2017). How to construct a mixed methods research design. *Kolner Z Soz Sozpsychol* 69 107–131. 10.1007/s11577-017-0454-1 28989188 PMC5602001

[B38] Schwaab B. Bjarnason-Wehrens B. Meng K. Albus C. Salzwedel A. Schmid J.-P. (2021). Cardiac rehabilitation in German speaking countries of Europe-evidence-based guidelines from Germany, Austria and Switzerland LLKardReha-DACH-Part 2. *J. Clin. Med.* 10:3071. 10.3390/jcm10143071 34069561 PMC8161282

[B39] Sember V. Meh K. Sorić M. Starc G. Rocha P. Jurak G. (2020). Validity and reliability of international physical activity questionnaires for adults across EU countries: systematic review and meta analysis. *Int. J. Environ. Res. Public Health* 17:E7161. 10.3390/ijerph17197161 33007880 PMC7579664

[B40] Spence J. C. Rhodes R. E. McCurdy A. Mangan A. Hopkins D. Mummery W. K. (2021). Determinants of physical activity among adults in the United Kingdom during the COVID-19 pandemic: the DUK-COVID study. *Br. J. Health Psychol.* 26 588–605. 10.1111/bjhp.12497 33336562

[B41] Stockwell S. Trott M. Tully M. Shin J. Barnett Y. Butler L. (2021). Changes in physical activity and sedentary behaviours from before to during the COVID-19 pandemic lockdown: a systematic review. *BMJ Open Sport Exerc. Med.* 7:e000960. 10.1136/bmjsem-2020-000960 34192010 PMC7852071

[B42] Symons M. Meira Cunha C. Poels K. Vandebosch H. Dens N. Alida Cutello C. (2021). Physical activity during the first lockdown of the COVID-19 pandemic: investigating the reliance on digital technologies, perceived benefits, barriers and the impact of affect. *Int. J. Environ. Res. Public Health* 18:5555. 10.3390/ijerph18115555 34067390 PMC8197021

[B43] Thomas E. Gallagher R. Grace S. L. (2020). Future-proofing cardiac rehabilitation: transitioning services to telehealth during COVID-19. *Eur. J. Prev. Cardiol.* Online ahead of print. 10.1177/2047487320922926 PMC7928989 33611474

[B44] Tschentscher M. Eichinger J. Egger A. Droese S. Schönfelder M. Niebauer J. (2016). High-intensity interval training is not superior to other forms of endurance training during cardiac rehabilitation. *Eur. J. Prev. Cardiol.* 23 14–20. 10.1177/2047487314560100 25404752

[B45] van Bakel B. M. A. Bakker E. A. de Vries F. Thijssen D. H. J. Eijsvogels T. M. H. (2021). Changes in physical activity and sedentary behaviour in cardiovascular disease patients during the COVID-19 Lockdown. *Int. J. Environ. Res. Public Health* 18:11929. 10.3390/ijerph182211929 34831684 PMC8623455

[B46] Vetrovsky T. Frybova T. Gant I. Semerad M. Cimler R. Bunc V. (2020). The detrimental effect of COVID-19 nationwide quarantine on accelerometer-assessed physical activity of heart failure patients. *ESC Heart Failure* 7 2093–2097. 10.1002/ehf2.12916 32696600 PMC7405478

[B47] Western M. J. Armstrong M. E. G. Islam I. Morgan K. Jones U. F. Kelson M. J. (2021). The effectiveness of digital interventions for increasing physical activity in individuals of low socioeconomic status: a systematic review and meta-analysis. *Int. J. Behav. Nutr. Phys. Act* 18:148. 10.1186/s12966-021-01218-4 34753490 PMC8576797

[B48] Williamson E. J. Walker A. J. Bhaskaran K. Bacon S. Bates C. Morton C. E. (2020). Factors associated with COVID-19-related death using OpenSAFELY. *Nature* 584 430–436. 10.1038/s41586-020-2521-4 32640463 PMC7611074

[B49] Yang Y. Koenigstorfer J. (2020). Determinants of physical activity maintenance during the Covid-19 pandemic: a focus on fitness apps. *Transl. Behav. Med.* 10 835–842. 10.1093/tbm/ibaa086 32926160 PMC7797716

