# Supplementary material for: Outpatient Cardiac Rehabilitation Closure and Home-Based Exercise Training During the First COVID-19 Lockdown in Austria: A Mixed-Methods Study
Source: Front Psychol. 2022 Feb 15;13:817912. doi: 10.3389/fpsyg.2022.817912 (PMC8887562; doi:10.3389/fpsyg.2022.817912)
Supplement: Supplementary file 1 [file Table_1.DOCX]

Supplementary Material

Supplementary Table 1. Interview schedule for semi-structured qualitative interviews

| Topic | Opening questions | Optional follow-up questions |
| --- | --- | --- |
| Comparing before and after the beginning of lockdown | What was it like for you during the past months, since the Coronavirus lockdown started in mid-March?  Before that, when had you last been to train at the center?  How did you used to train, do sports, or be physically active before the Coronavirus situation?  What has it been like with regard to physical activity, training, or coronary sports since the beginning of the lockdown?  In your estimation, have you been able to keep up the recommended amount of physical activity or training since mid-March?  In your estimation, how has your physical fitness and your heart health developed since March? | Have you been able to do sports?  What were you able to do?  What were you unable to do?  Did the lockdown / time in home office for you impact on possibilities for training?  Did the changed daily routines (e.g., more or less flexibility) during lockdown / time in home office for you impact on possibilities for training?  How did changes in your social life (e.g., more or less contact to family members and friends) during lockdown / time in home office for you impact on possibilities for training? |
| Photovoice^a^ | Can you talk about the photos you brought with you? | What is the significance of this photo for you with regard to physical activity during lockdown? |
| Reflections on individual clinical test results^b^ | What are your thoughts, looking at these test results? | If you think about different measures for keeping a heart-healthy lifestyle, what has worked well for you in the past months?  What has not worked so well? |
| Support with physical activity | What would you say that you have missed most since mid-March, or what would you say you would wish for most (with regard to physical activity or training)? | What would you say that you personally have found most helpful, or what would you also recommend to others (with regard to physical activity or training)? |
| Digital technologies to support physical activity | We have a possibility to explore, how digital technologies could support you at home, for example with physical activity or training. Have you ever used a digital technology to support you with physical activity or sports? | Digital technologies include: internet, computer and online websites; smartphone and Apps you use on your smartphone; smart TV; smart watch, sensors, fitness trackers (e.g., wrist worn devices), etc.  What is your attitude in general regarding digital technologies?  Would you have any suggestions or ideas, how such technologies could be used to support you at home?  If you think back over the past few months since mid-March, was there any particular reason or opportunity to use such technologies?  Did you perhaps deliberately start using such technologies because of the Coronavirus restrictions? |
| Concluding questions | Apart from what we already talked about, is there any other question you wish I would have asked you?  Would you have any further thoughts or comments on the topic which you feel are important? |  |
| ^a^ In an adapted photovoice activity, participants were invited to bring anonymized photos to the interview. Participants were asked to select up to three photos which for them symbolized important experiences related to physical activity during the Coronavirus lockdown.  ^b^ At this point in the interview, the interviewer showed and explained to the participant the clinical test results pre and post lockdown. | | |
